# The multifactorial complexities of autoimmune development in Pemphigus vulgaris: Critical evaluation of the role of environmental and lifestyle “exposome” factors

**DOI:** 10.3389/fimmu.2022.1058759

**Published:** 2023-01-10

**Authors:** Olumayowa T. Adebiyi, Dominique F. Galloway, Michael S. Augustin, Animesh A. Sinha

**Affiliations:** Department of Dermatology, Jacobs School of Medicine and Biomedical Sciences, Buffalo, NY, United States

**Keywords:** pemphigus vulgaris (PV), induction, trigger, environmental factors, exacerbation, lifestyle factors

## Abstract

Pemphigus vulgaris (PV) is a potentially life-threatening blistering disorder characterized by autoantibodies directed against cell-cell adhesion molecules that serves as an excellent model to study human autoimmune development. Numerous studies have identified specific Human Leukocyte Antigen (HLA) genes, in particular DRB1*0402 and DQB1*0503, that confer disease risk. Although HLA is required, it is not sufficient for the initiation of disease. As with all autoimmune diseases, the etio-pathogenesis of PV is complex, meaning it is multifactorial. Susceptibility is polygenic, and the search for non-HLA disease-linked genes continues. Moreover, twin studies across autoimmune conditions indicate that non-genetic environmental and lifestyle factors, which can be collectively grouped under the term “exposome”, are also major contributors to disease development. The literature presents evidence for the potential role of multiple triggers such as medications, infections, stress, diet, immunizations, and sleep to influence the etiology, pathophysiology, and prognosis of PV. However, a clear understanding of the degree to which specific factors impact PV is lacking. In this investigation, we comprehensively review the environmental elements listed above and consider the strength of evidence for these factors. The overall goals of this work are to provide greater insights into the factors that influence disease susceptibility, disease development and disease course and ultimately help to better guide clinicians and inform patients in the management of PV.

## 1 Introduction

Pemphigus vulgaris (PV) is the most common subtype of a group of rare autoimmune blistering disorders (AIBD) in which autoantibodies primarily directed against the cell-cell adhesion molecules desmoglein 3 (Dsg3) and desmoglein 1 (Dsg1) lead to characteristic epidermal blistering. PV is classified into the major subtypes of mucosal-dominant, and mucocutaneous. The existence of a cutaneous phenotype of PV has also been documented in several case reports and recent studies (1–4). The etiology of PV is clearly multifactorial involving both genetic and environmental factors. Although key Human Leukocyte Antigen (HLA) genes, in particular DRB1*0402 and DQB1*0503, have been identified, we have little to no information on the broader genetic architecture that undergirds disease susceptibility. Beyond the holes in our information on polygenic risk elements, we similarly have a weak understanding of environmental factors that trigger or influence disease development and clinical expression in PV. Here, we undertook a comprehensive review by searching PubMed/Medline databases to determine the level of existing support for the relationship between PV and multiple environmental and lifestyle factors that contribute to the broader “exposome” including: medications, infections, stress, diet, immunizations, and sleep.

In this investigation, we comprehensively evaluated the evidence for, and the level of support associated with each of the factors listed above. We sought to illuminate the relationship more clearly between these factors and the onset, recurrence, or exacerbation of PV. This information deepens our understanding of disease risk as well as the basis of phenotypic variation and disease heterogeneity; and provides a step forward towards a more detailed framework to support disease relevant decision-making by physicians and patients.

## 2 Approach to review and methods

To accomplish these goals, we performed a comprehensive literature review by utilizing the PubMed/Medline database to identify key articles related to the onset and/or exacerbation of pemphigus vulgaris. We conducted a search within the parameter of a 20-year submitted literature period from January 2001 to December 2021. Search strings were compiled per each factor and combined to encompass specific keywords (**Table 1**). The inclusion criteria involved articles that reported or reviewed cases relating to PV induction, trigger and or exacerbation by environmental and or lifestyle factors under infection, medication, stress, immunization, diet, and stress. The exclusion criteria involved articles that reported cases of pemphigus foliaceus, or other pemphigus subtypes, and articles that were not in English. Initial results of the literature search based on the inclusion and exclusion criteria outlined yielded *n =* 2093 papers. A total of *n =*1460 duplicates were identified. All duplicates were excluded from this study. *n=* 39 met additional exclusion criteria, resulting in *n* = 672 papers that met inclusion criteria. All articles meeting inclusion criteria were then further screened in a two-step process:

**Table 1:** Keyword terms utilized in database search string for Term 2.

Medication	Infection	Stress	Diet	Immunization	Sleep
Medication Pharmaceutical Preparations/adverse effects Dietary Supplement/adverse effects Therapeutics/adverse effects Sulfhydryl compounds/adverse effects Penicillin Rifampin Thiol Non-thiols	Symptom Symptom flare up Illness infections/etiology infections/adverse effects	Stress Psychological Trauma Chronic stress Depression Anxiety Stressor Mood Mood disorder Emotion Emotional Psychiatric Distress Sickness behavior Stress, psychological/complications	Diet Food Nutrition Glucose Nutrient Diet, food and nutrition	Immunization Vaccines injection inoculation serum	Sleep sleepy fatigue lethargy rest REM NREM Slow wave sleep SWS somnolence Sleepiness

Step 1: Screening of title & abstract on PV induction/exacerbation, which yielded *n* = 186 papers of relevance to overall study goals.

**Figure 1:**
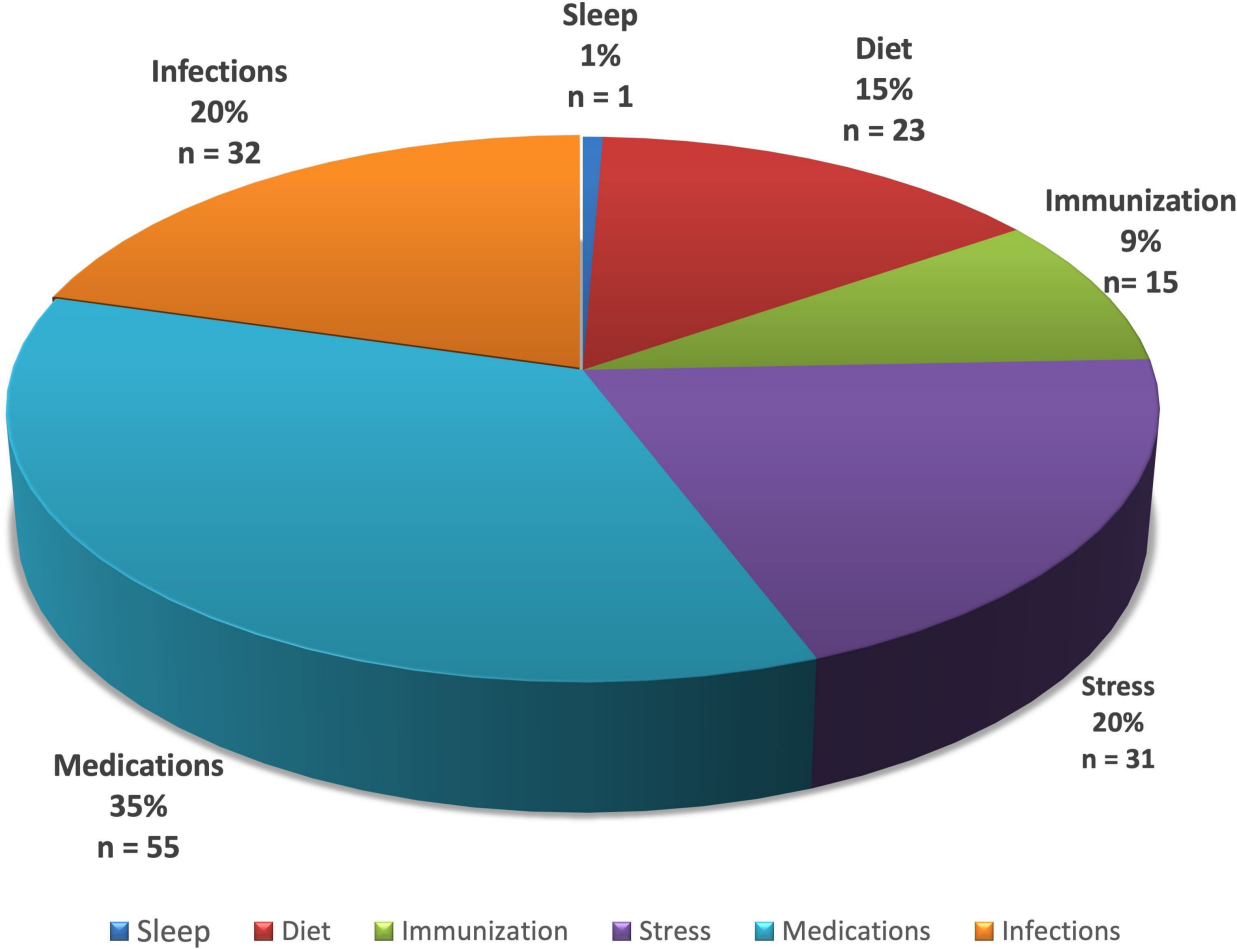
Distribution of literature results after screening for PV induction or exacerbation as linked to specific lifestyle/environmental factors. Subgroups identified are represented as a percentage (%) and number (n) of all studies meeting criteria.

Step 2: Screening of content relevant in Step 1 that provides evidence for the potential role of environmental and lifestyle “exposome” factors on PV pathophysiology, yielded *n =* 157 papers (**Figure 1**).

After the 2-step screening process, we undertook a detailed manual assessment to determine the relationship of each one of the environmental/lifestyle exposome factors listed above to the etio-pathogenesis of PV.

## 3 Findings on environmental and lifestyle “exposome” factors

Support for the role of medications in pemphigus was found to be the most highly represented in the literature relative to the other factors studies (**Figure 1**). The role of sleep was least reported. It should be noted that the underlying disease relevance of each factor studied may not be reflected only by the number of associated articles in the literature, which may simply be the result of investigative biases and access at this point in time. More extensive data in larger patient populations and future mechanistic studies will be required to better establish the relative rank of exposome linked factors in terms of contributing to disease development and clinical modulation.

### 3.1 Medications

Medications known to induce or exacerbate PV flares have been studied extensively in the literature (see **Table 2**). Drug induced pemphigus (DIP) has been recognized as a distinct subtype of pemphigus distinguishing this known etiology from the idiopathic form. DIP makes diagnosis challenging due to the similarities in clinical, histological and immunochemistry features shared with idiopathic PV. The earliest reports of DIP were first described by Degos et al. in 1969 (51). Isolated case studies have provided clarity regarding the specific drugs that may cause pemphigus foliaceus (PF) and/or pemphigus vulgaris and those that have been reported to induce and/or exacerbate PV flares.

**Table 2:** Studies in support of the hypothesis that PV is triggered or exacerbated by medications.

Drug Category	Proposed Pathomechanisms	Drug	# of publications in support	Study type	Model	References
Thiols	•Stimulate antibody production and increase antigenicity of desmoglein proteins (5–8)	Bucillamine	4	Review Case Report, Review Review	- Human - -	(9) (10) (11) (8)
		Captopril	3	Case-Report, Original research, Longitudinal	Human Human Human	(12) (7) (13)
		Gold Sodium thiomalate	3	Review^*^ review^*^ Review^*^	- - -	(14, 15),^*^ (8, 11),^*^ (16, 17),^*^
		Penicillamine	3	Case report Review^*^ Review^*^	Human - -	(18) (6, 14),^*^ (6, 14, 19),^*^
		Penicillin	3	Review^*^ Review^*^ Review^*^	Human - -	(15, 20),^*^ (14, 21),^*^ (16, 17),^*^
		Piroxicam	2	Review^*^ Review^*^	- Human	(6, 14),^*^ (16, 22),^*^ (6),^*^ (23),^*^
		5-thiopyridoxine^*^	1	Review^*^	–	(16, 23),^*^
		Amoxicillin/clavulanic acid	1	Case-Report	Human	(24)
		Cephalosporin (Cefixime) (Ceftazidime)	1	Case-report	Human	(13)
		Pyritinol^*^	1	Review^*^	Human	(16, 23),^*^
		Thiopronine	1	Review^*^	Human	(16, 23),^*^
Phenols	•Inhibit enzymes responsible for keratinocyte aggregation (16, 25) •Disrupt thiol-cysteine binding epithelial cells (16, 23, 25) •Activate plasminogen activators which disaggregate keratinocytes and increase synthesis of cytokines (16, 23, 25)	Rifampin	6	Case-Report, Case report Review^*^ Review Review	Human Human - - -	(11) (26) (16, 17),^*^ (26) (11, 26),
		Levodopa	2	Review^*^ Review^*^	Human -	(11, 17),^*^ (16, 17),^*^
		Aspirin	1	Review^*^	Human	(16, 17),^*^
		Cefadroxil	1	Review^*^	Human	(16, 17),^*^
		Pentachlorophenol	1	Review^*^	Human	(16, 17),^*^
		Phenobarbital	1	Review^*^	Human	(16, 17),^*^
Non-thiols & non-phenols	•Stimulate keratinocytes to produce proinflammatory cytokines such as TNF and IL-1 (19, 27–29) •Activate proteases and complement that contribute to acantholysis (19, 27–29)	Glibenclamide	3	Review Case-report Review^*^	- Human -	(20, 30) (30) (11, 15),^*^ (21),^*^
		Carbamazepine	2	Case report Case report	Human	(31, 32)
		Chloroquine/hydroxychloroquine	2	Review^*^ Case report	- Human	(6, 33),^*^ (33)^*^
		Cilazapril	2	Review^*^ Case report	- Human	(20, 27),^*^ (27)^*^
		Fosinopril	2	Review Case report	- Human	(30) (34)
		Imiquimod	2	Case-report Case-report	Human	(35) (36)
		Ingenol mebutate	2	Review Case Report	- Human	(6) (37)
		Acetazolamide	1	Case-Report	Human	(38)
		candesartan	1	Case-report	Human	(39)
		Ciprofloxacin	1	Case report	Human	(40)
		Cocaine	1	Case-report	Human	(41)
		Diazinon	1	Review^*^	-	(20, 42),^*^
		Dipyrone	1	Review*	Human	(20, 43),^*^
		Hydrochlorothiazide	1	Case-report	Human	(28)
		Irbesartan	1	Case report	Human	(28)
		Lisinopril	1	Case-report	Human	(44)
		Methylisothiazolinone	1	Case-report	Human	(29)
		Metoprolol	1	Case-report	Human	(45)
		Phenytoin	1	Case-report	Human	(32)
Immuno-therapy	•Pathomechanism is not fully understood	Nivolumab	2	Case report Case report	Human Human	(46) (47)
Cancer therapy	•Increase the antigenicity of keratinocytic surface molecules via peptidyl sulfhydryl disruptive mechanisms (12, 13)	Radiotherapy	6	Review Case Report, Case-report, Case report Case report Case report	Human	(6) (42) (48) (49) (49) (50)

^*^Indicates a review paper that references literature out of our literature search range (2001-2021).

Various case reports and *in vitro* experiments since the earliest findings of DIP report similar findings and have identified drugs with certain chemical properties as the most common triggers of induced PV. The concept of drug induced PV is by far the most common environmental factor linked to the induction of pemphigus (see **Figure 1**). The literature provides evidence of reported cases of DIP following therapy for a non-related pemphigus diagnosis and the eventual rapid remission of PV upon discontinuation of the culprit agent (12, 20, 25, 52). The most common clinical variant associated with drug exposure is PF, however, PV has also been described. The drugs linked to DIP vulgaris are categorized into 3 main categories: thiol drugs, phenols, and non-thiols/non-phenols (16).

Thiol drugs contain a sulfhydryl group (-SH) and are responsible for the stimulation of antibody production through interactions that increase antigenicity of desmoglein proteins. Experiments done by Ruccco et al. demonstrated the induction of acantholysis without antibody mediation (5). The main thiol containing PV inducing drugs include penicillamine, bucillamine, and captopril (6, 7, 9, 10, 12, 14, 16, 18–20, 22, 53). Other known thiol drugs listed in **Table 2** have been identified in isolated in case reports, longitudinal studies, and reviews (6, 8, 11, 14–17, 20–24, 54).

The most reported phenol containing medications known to induce PV include Rifampin, Cefadroxil, Aspirin, Levodopa, Pentachlorophenol and phenobarbital (11, 16, 17, 20, 26). The mechanisms of induction proposed by Brenner et al. are similar to those with that of the thiol drugs (25). The proposed mechanism of acantholysis caused by thiol and phenol drugs is as follows: inhibition of enzymes responsible for keratinocyte aggregation, disruption of thiol-cysteine binding epithelial cells, activation of plasminogen activators which disaggregate keratinocytes and increase synthesis of cytokines (13, 16, 53).

Interestingly, some studies have highlighted an increased incidence of PV induction by non-thiols rather than thiols. This difference goes beyond the chemical nature of the causative agent; differences regarding the mechanism of induction is also key to understanding the pathophysiology of PV (9, 11, 14, 30, 31, 42, 43, 53). It is important to know what specific chemical group, and the pemphigus subtype being induced by said drug. Non-thiol drugs are mainly identified by sulfur and amide components. Most of the commonly prescribed diuretics fall under this category. Known drugs include Cilazapril, fosinopril, lisinopril, nifedipine, imiquimod, carbamazepine, chloroquine/hydroxychloroquine, dipyrone, glibenclaimde, ingenol mebutate, acetazolamide, candesartan, ciprofloxacin, cocaine, diazinon, hydrochlorothiazide, irbesartan, methylisothiazolinone, metoprolol, phenytoin (11, 20, 27–29, 32–41, 44, 45). These groups (sulfur and amides) are postulated to induce PV *via* immunological mechanisms that stimulate keratinocytes to produce proinflammatory cytokines such as TNF and IL-1 (8, 55–57), leading to the activation of proteases and complement that contribute to acantholysis.

To our knowledge, reports of PV induction following radiotherapy and immunotherapy are rarely reported in the literature. Isolated case-control studies identify an elevated risk of developing PV with previous radiotherapy (RT) and ultraviolet light (42, 48–50, 55, 58–60). These studies report an accompanying malignancy, most commonly breast cancer or lymphomas, with an associated eruption of pemphigus lesions that range from 1 week to 1 year following irradiation. A case-report revealed the induction of PV 1 month following radiation for hypopharynx carcinoma and other related neoplastic RT-induced pemphigus (61). The mechanisms responsible for RT induced pemphigus share some similarities to that of DIP. Specifically, ionizing radiation is said to increase the antigenicity of keratinocytic surface molecules *via* peptidyl sulfhydryl disruptive mechanisms (42, 48, 49, 59). There are only two reported cases of DIP developed in a patient after treatment with the immunotherapeutic agent nivolumab (46, 47).

### 3.2 Infections

Several studies have looked at the Herpesviridae family of viruses (HSV) that produce various skin/oral lesions or ulcerations including “cold sores”. Transmission of this viral family is usually *via* skin-to-skin contact or contact with bodily fluids of an infected individual. Potentially, lesions caused by HSV could lead to the exposure of intraepidermal epitopes to the immune system causing intercellular adhesion molecule antibody production, and thus the development of PV (62–64). In a paper by Senger et al., the link between PV and the various Herpesviridae was explored by evaluating levels of antiviral antibodies. Anti-HSV1 antibody levels were found to be higher in active PV patients than in remittent patients and controls, supporting a potential role of HSV1 in disease expression and clinical activity. However, there was no way to establish causality in this retrospective evaluation, and further studies would be required to follow anti-HSV levels in patients longitudinally. Alternatively, HSV could simply mimic PV like erosions and/or contribute to the growth of preexisting lesions rather than initiating autoimmunity. This same paper examined the literature in terms of relations between Cytomegalovirus (CMV) and Varicella Zoster virus (VZV) and PV; there were no substantial relationships found (65).

Bacterial infections such as *Legionella pneumophila*, *Staphylococcus aureus*, *Proteus Vulgaris*, and *Pseudomonas Aeruginosa* have also been studied for potential associations with PV (see **Table 3**). *Staphylococcus aureus* is a gram-positive bacillus that can cause both toxin mediated and systemic infections in a host. One of the presentations of *Staph aureus* is Scalded Skin Syndrome that is mediated by the exotoxins A and B. It has been identified that exotoxin A targets and cleaves the Dsg-1 protein which leads to loss of cell-cell adhesion, mimicking the autoimmune condition seen in PF (82, 90). *Legionella pneumophila* is a gram-negative bacterium that is known to cause Legionnaires’ disease. In a study by Tirado et al., the relationship between the prevalence of legionella specific antibodies and PV patients was examined. This study concluded that the antibodies themselves may be the trigger for autoimmunity (84). This is in opposition to other studies that tend to equate the infection being secondary to immunosuppressive therapy. Examples of bacteria that support the latter include *Pseudomonas aeruginosa* and *Proteus Vulgaris* (79, 91).

**Table 3:** Studies in support of the hypothesis that PV is triggered or exacerbated by infections.

Infection:	Proposed Pathomechanisms	Organism	# of publications in support:	Study type:	Model:	References:
Herpesviridae	•Exposure of intraepidermal epitopes to the immune system (62–64)		17	Case report Letter to editor Review Basic Sciences Letter to editor Case report correspondence Clin. & Lab. Inv Case report Prelim. Report correspondence Report Case report Case report Case report Case report^*^ Basic Sciences	Human	(63) (66) (65) (67) (64) (68) (69) (70) (62) (71) (72) (73) (74) (75) (76) (77)^*^ (78)
Bacteria	•Mechanism is not fully understood •Nocardia can disseminate to the skin, possibly leading to exacerbation of PV (79)	Nocardiosis	1	Case report	Human	(79)
	•Molecular mimicry (80) •Epitope spreading (80) •Unmasking of hidden antigens^ ^ (80)	Mycobacterium	1	Correlational Study		(80)
	•Pathomechanism is not fully understood.^ ^ (81)	H. Pylori	1	Report		(81)
	•Exotoxin A targets and cleaves the Dsg-1^ ^ (82)	Staph. Aureus	1	Retrospective study		(83)
	•Antibodies for Legionella pneumophila (84)	Legionella Pneumophila	1	Correspondence		(84)
Fungal	•Pathomechanism is not fully understood. (85)	Candida	1	Basic Sciences	Human	(85)
COVID-19	•Molecular mimicry (86 ^*^) •Bystander activation (86 ^*^) •Epitope spreading (86 ^*^) •Combination of these 3 autoimmune phenomena (86 ^*^)		4	Case reports Case report Comment Letter to the editor^*^	Human	(87) (88) (89) (86)^*^

^*^Indicates a review paper that references literature out of our literature search range (2001-2021).

Fungal infection such as oral *Candidiasis*, *Aspergillus*, and *Pneumocystis jiroveci* may play a role in the exacerbation of Pemphigus vulgaris due to their opportunistic tendency in patients on immunosuppressive therapy (90), but there is scant literature to establish their role they play in autoimmunity.

More recently several case reports on COVID-19 and PV have appeared. Ghalamkarpour et al. reported a patient who was previously diagnosed with Pemphigus vulgaris went into remission but had exacerbation following a Covid-19 infection (87). Interestingly, De Medeiros VLS et al., presented a case of a previously healthy patient who after contracting COVID-19 presented with bullae on his chest that was determined to be PV (88). There have also been reports that SARS-CoV-2 can lead to autoimmunity and hence the induction of cutaneous diseases. Further research must be done to determine the actual role that COVID may play in the development and/or exacerbation of PV.

Another recent interesting study shed a light on a potential link between roseolovirus and autoimmunity. In a study done by Bigley et al., a mouse model was infected with murine roseolovirus, which is related to human roseolovirus, and it was hypothesized that murine roseolovirus (MRV) impacted central tolerance by disrupting medullary thymic epithelial cells (mTECs) and CD11c+ thymic dendritic cells (tDCs) in the thymus. The authors were able to determine that neonatal MRV infection leads to a variety of autoantibodies in adult mice (92). This mechanism of action has not been previously proposed in the context of pemphigus vulgaris development but lends support to extend the investigation into the viral induction of autoimmunity.

### 3.3 Stress

Stress and stressful life events have long been postulated as potential triggering factors for skin disease. Stress can be defined as a state of emotional or physical tension that induces the release of stress hormones, such as adrenaline and cortisol. This initial hormonal response is referred to as “fight or flight” and it serves as a survival mechanism to react quickly to a perceived threat. While acute stress can be beneficial to the individual in the context of dangerous situations, chronic stress where hormonal levels remain elevated far longer than is necessary for survival can have deleterious consequences for health.

The skin and the central nervous system are both derived from the embryonic ectoderm, which may explain the existence of a relationship between psychological factors and dermatologic diseases (93). In addition, there has been growing evidence of a unique neuroimmunocutaneous system. The skin, nervous system, and immune system all share hormones, cytokines, and neurotransmitters as a way to communicate, which can account for a pathogenic link between stress and the onset or worsening of autoimmune skin diseases, such as pemphigus vulgaris (PV) (14).

Some of the notable stressful life events that are most associated to PV include environmental disasters, war, terrorism, partner’s or near relative’s death, separation from partner, physical trauma, sexual aggression, or sex-related disturbance (14). The first two cases of PV occurring after a stressful event were reported by Brenner and Bar-Nathan in 1984 (94). Since this initial observation, a limited number of studies have also pointed to stress as an inducing and triggering factor in the etiology of PV. In a clinical investigation involving 13 pemphigus patients with personality disorders, it was revealed that 12 out of 13 patients had experienced a stressful event during the year preceding the onset of the autoimmune disorder (14). A combined retrospective and prospective epidemiological study evaluated all cases of pemphigus from 2000 to 2004. It was concluded that all 10 patients that participated in the study had recordable stressful life events less than 6 months before their first clinical symptoms or worsening of pemphigus (94). In an isolated study, two exogenous factors were found to trigger PV in a 56 year-old Jewish woman of Ashkenazi origin. This woman experienced the Holocaust and the Persian Gulf War, and it was concluded that both emotional stress and the drug rifampin led to her first clinical symptoms of PV (26).

Another study had the aim to establish incidence of acquired bullous dermatitis (BD) among hospitalized patients in Eastern Croatia before and after war. There was a higher incidence of acquired BD during the years of war and the period immediately after compared to before the war. It was concluded that prolonged exposure to stressful conditions influenced the incidence of disease (95). A case-control study had the objective to estimate the initial serum levels of TNF-alpha in pemphigus patients and compare them with history of stress, body surface area affected, disease severity, and disease outcome. Significantly higher serum levels of TNF-alpha were found in PV patients compared to healthy patients. 30% of the PV patients reported severe emotional stress within a month prior to the onset of disease. Those patients had high initial levels of serum TNF-alpha and showed poor response to treatment, resulting in poor prognosis (96).

A bidirectional relationship between PV and psychological stress has also been reported. There have been several reports of PV impairing patients’ quality of life (97–103), which can then impact one’s stress levels and mental health (see **Table 4**). Some forms of psychological distress could also be in part due to one’s perception of his/her own body image (104). In multiple investigations, there was a significant association between pemphigus and an increased risk of depression and/or anxiety (106). In a case-control study, 30 PV patients and 30 healthy patients were interviewed for their health-related quality of life (HR-QoL) and psychological profile. Anxiety and depression were found in 60% and 50% of the PV patients. The persistence of a poor HR-QoL and higher levels of anxiety and depression is also considered as a risk factor for a relapse of the disease because the onset of anxiety and depression has been associated with immune system dysregulation (105). Thus, it is possible that stress could both play a role in triggering disease, and also that the disease state itself can generate stress that not only impacts quality of life, but potentially perpetuates stress linked autoimmune mechanisms to propagate disease activity and clinical flares. It is commonly recommended that PV patients receive consistent psychiatric assessment and intervention as part of a treatment plan to prevent an exacerbation of the disease.

**Table 4:** Studies in support of the hypothesis that PV is associated with a stressor.

Stressor	Proposed Pathomechanisms	# of publications in support	Study type	Model	References
PV impacting mental stress/QoL	•Psychological distress due to one’s perception of his/her own body image (104) •Poor HR-QoL and higher levels of anxiety and depression is a risk factor for a relapse of the disease (105)	15	Qualitative study, Cohort study, Case-control, Case-control, QoL, Cross-sectional study, QoL, Questionnaire, Cross-sectional study/Questionnaire, Questionnaire, QoL questionnaire, QoL questionnaire, QoL questionnaire, QoL, Review	Human	(102) (106) (107) (105) (101) (108) (100) (104) (109) (99) (98) (97) (110) (103) (111)
Psychological/ Emotional Stress inducing PV	•Elevates levels of TNF-alpha can propagate disease activity (96)	12	Review^*^ Review Review^*^ Review^*^ Review^*^ Retrospective and prospective epidemiological study Case report Case control Retrospective epidemiological study Case-control questionnaire, Review^*^ Review	Human	(20, 94),^*^ (112) (14, 113),^*^ (93, 94),^*^ (113, 114),^*^ (115) (26) (96) (95) (116) (113, 117),^*^ (111)
Physical stress/trauma inducing PV	•Stimulates epitope spreading and antigen presentation (118)	7	Case-control Review Case series Retrospective case-control study Case report Review^*^ Retrospective study	Human	(119) (112) (118) (120) (121) (117, 122),^*^ (123),^*^ (124),^*^ (125)

^*^Indicates a review paper that references literature out of our literature search range (2001-2021).

In addition to psychological stress, there are studies that report on the impact that physical stress has on the exacerbation of pemphigus vulgaris as well. In a case series, a total of 36 PV patients had a history of physical trauma before the onset of lesions (118). In a retrospective study of 15 PV patients, sites of dental trauma-induced lesions were detected in 13 patients (125). A case report of a 49-year old woman described the development of PV after a Mohs surgical excision of squamous cell carcinoma. The pre-operative lesion did not reveal PV, however, the postoperative lesion revealed PV without any residual squamous cell carcinoma. The report concluded that Mohs surgery, and perhaps other surgical interventions, may activate PV (121). UV radiation can also induce or exacerbate the clinical manifestation of PV, in addition to physical factors, such as x-ray radiotherapy, burns, major surgery, and cosmetic procedures (117). A retrospective case-control study revealed that approximately 40% of patients had been continuously exposed to UV radiation in their work 5 years prior to developing the disease. The distribution of PV lesions on sun-exposed areas also supports these findings (120). The exposure to the sun and other UV sources suggests special caution for PV patients because of the risk for photo-induced relapses (112). Taken together, there is literature to indicate that various forms of physical trauma to the skin or mucosa can trigger PV, therefore, unnecessary operations should also be avoided or postponed (118).

There appears to be strong support for the essential role of stress in the etiology of pemphigus vulgaris *via* psychosomatic mechanisms. However, it remains difficult to draw significant conclusions due to the relatively limited number of research studies, small sample sizes, and lack of controlled studies. Additional studies are needed to clarify whether psychological stress is an inducing factor of PV, a complication of the disease, or an adverse effect of its therapy.

### 3.4 Diet

Nutrition has been well documented as an exogenous factor influencing several disease states. There is growing research on the relation between nutrition and specific skin diseases, such as atopic dermatitis and urticaria (several foods), dermatitis herpetiformis (gluten), and porphyria cutanea tarda (alcohol) (126). Due to the complexity of autoimmune skin disease, its clinical course and the variability of human nutrition, dietary factors in relation to PV have remained elusive. However, recent epidemiological, clinical, and experimental data collected have allowed the inclusion of nutrition as an agent that can impact PV.

Many studies have reported on the possible induction of PV by dietary ingredients rich in thiols, phenols, and tannins (14, 16, 20, 112, 114, 117, 120, 127–132). Similar to medications that contain thiol groups, dietary sources high in similar compounds have been reported as triggering factors. Some of these foods and drinks include many fruits, garlic, onions, leeks, spices, legumes, nuts, tea, red wine, and beer (16, 128). Some of these ingredients are also widely consumed in India and Brazil, which might, at least partially, explain the high incidence of pemphigus in Indian and Brazilian patients (16). In addition, the high incidence of PV in Russian Jews has been linked to the frequent use of spices in this ethnic group (14). In an isolated study, a woman from Naples had an abrupt outbreak of pemphigus following a meal heavily seasoned in garlic (thiol-containing) after years in remission. A 49 year old man who consumed large amounts of garlic developed superficial pemphigus and the lesions dissipated while on a garlic-free diet. Another woman from Poland had remission of the disease after withdrawing large amounts of leeks from her diet (16). These reports highlight individual cases of onset or exacerbation, however, in order to better understand the potential role of these compounds in the pathogenesis of pemphigus, controlled studies are needed.

In one study, 40 volunteers were divided into four groups to measure the presence of tannic acid in the skin of different populations. The group that had a high dietary tannin intake correlated with higher levels of tannins in the skin (127, 128). In another study conducted by Brenner, five skin explants were cultured with tannic acid at different concentration levels and the most constant and specific induced effect was marked by acantholytic changes (128). PV patients living in Amazonian, Mediterranean, and Indian subcontinent areas where the diet is rich in tannins should be informed about tannins as a possible trigger (130). The suggested mechanisms for thiol-induced acantholysis include the direct biochemical impairment of cell adhesion, protease activation, and immunological reaction with the formation of neo-antigens. The suggested mechanisms for phenol-induced PV include the release of IL-1a and TNF-a from keratinocytes by phenol molecules, which can trigger cutaneous inflammation. These two cytokines enhance the synthesis and regulation of complement and proteases, such as C3 and plasminogen activator, which have been associated to the pathogenesis of acantholysis in PV (127, 128).

There have also been reports of other dietary sources that may induce PV (see **Table 5**). In one investigation, the findings suggest that the intake of a dietary walnut antigen through gastrointestinal epithelial cells can activate naive B cells in subjects genetically predisposed to PV through a “hit-and-run” mechanism. According to this mechanism, the cross-reactivity between an infectious antigen and autoantigen can lead to a long-lasting immune response, even once the pathogen is cleared, because the continued presence of the autoantigen would perpetually drive subsequent autoantibody generation and the development (and perpetuation and/or exacerbation of) disease (144). In a case report, it was proposed that the immune-enhancing effects of herbal supplements, specifically Echinacea and the alga Spirulina platensis, contributed to flares of pemphigus vulgaris in two patients. It was suggested that increased production of TNF-alpha may be playing a role in disease exacerbation, although additional research is required to confirm this mechanism (145).

**Table 5:** Studies in support of the hypothesis that PV is triggered or exacerbated by a nutritional element.

Nutritional Element	Proposed Pathomechanisms	# of publications in support	Study type	Model	References
Phenols, thiols, tannins	•Triggers direct biochemical impairment of cell adhesion, protease activation, and immunological reaction with the formation of neo-antigens (127, 128) •IL-1alpha and TNF-alpha from keratinocytes trigger cutaneous inflammation (127, 128)	13	Review Review^*^ Prospective exposure study Review^*^ Review Review Review E-survey Review^*^ Retrospective case-control study/questionnaire Review^*^ Cross-sectional study Review	Human	(20) (14, 133),^*^ (127) (16, 126),^*^ (134),^*^ (128) (112) (114) (129) (130), (135)^*^ (136),^*^ (137),^*^ (120) (117, 137),^*^ (131) (132)
Micronutrients and trace elements	•Surplus and deficiency impact the functioning of the immune system, wound healing, and antioxidant defense (138)	7	Review Case-control Review Case-control Case-control Case-control Case-control	Human	(20) (139) (140) (138) (141) (142) (143)
Walnut	•Activates naive B cells through a “hit-and-run” mechanism (144)	1	Case-control	Human	(144)
Herbal supplements	•Increases production of TNF-alpha (145)	1	Case reports	Human	(145)
Dieting	•Pathomechanism is not fully understood.	1	Case-control questionnaire	Human	(116)
Fast food	•Pathomechanism is not fully understood.	1	Case-control	Human	(146)

^*^Indicates a review paper that references literature out of our literature search range (2001-2021).

In regards to micronutrients, one study found that serum vitamin D levels are significantly lower in newly diagnosed PV patients compared to healthy controls. There was also a negative correlation between the vitamin D level and the severity of disease. Vitamin D is known as an important immunomodulatory agent and it was suggested that the insufficient vitamin D level could be considered as an environmental factor that contributes to the pathophysiology of disease (141). Recently, the possible beneficial role of retinoic acid has also been discussed (147). In another case-control study, the results show that PV causes depletion of some trace elements including zinc, selenium, and copper. These may have important roles in the functioning of the immune system, wound healing, and antioxidant defense, so supplementation could potentially alleviate disease severity and mortality. But again, clinical trials are needed to confirm this theory (138).

Another study found that copper concentrations in Iranian patients with PV were less than in controls (140). An investigation on the trace element profile of pemphigus patients in Southeastern Brazil showed that PV patients had higher lead (Pb) values as compared to the controls. Pemphigus is endemic in Southeastern Brazil and Pb is known to play an immunomodulatory role that favors Th2 proliferation and consequent production of Th2 cytokines. Pb contamination in chronic doses may constitute a trigger factor for PV pathogenesis (139).

Taken together, the relevance of nutritional factors seems to be underestimated in the induction of PV. Avoiding exposure of genetically predisposed individuals to ingredients high in thiols, phenols, and tannins may be beneficial in the prevention and management of PV. However, there are numerous dietary factors that need to be more fully investigated. In the future, modulation of nutrient and micronutrient levels in PV patients may be part of a viable management strategy (20).

### 3.5 Immunization

Recent attention has been given to COVID-19 vaccination and Pemphigus vulgaris (see **Table 6**). There were two cases of patients who received the Pfizer COVID-19 vaccine. Both patients, who were previously healthy, later developed oral lesions and blistering on their trunks and had demonstrated Dsg3 and Dsg1 autoantibodies (148, 149). Damiani et al. report multiple cases of patients forming bullae on their back, trunk, arms, and legs after the first shot of the Pfizer and Moderna COVID-19 vaccines. They proposed that both mRNA vaccines may trigger relapses in PV patients (150).

**Table 6:** Studies in support of the hypothesis that PV is triggered or exacerbated by immunizations and vaccinations.

Immunization	Proposed Pathomechanisms	# of publications in support	Study type	Model	References
COVID-19	•Induces autoimmunity by molecular mimicry (148)	5	Case report Case report Letter to editor Case reports^*^ Review^*^	Human	(148) (149) (150) (151)^*^ (152)^*^
Influenza	•Hyperimmune reaction (153) •Cross reaction of vaccine antigens with pemphigus antigens (153)	1	Case report	Human	(153)
Anthrax	•Hyperimmune reaction (154)	1	Case report	Human	(154)
Hepatitis B	•Molecular mimicry theory (155) •Nonspecific activation of the immune system promoting the activation of autoreactive T cells (155)	1	Case report	Human	(155)
Shingrix Vaccine	•Hyperactive immune (156) •Antigenic vaccination (156) •Genetic predisposition (156)	1	Review	Human	(156)

^*^Indicates a review paper that references literature out of our literature search range (2001-2021).

Another well-known vaccine that may have led to the reactivation of Pemphigus Vulgaris is the influenza vaccine, which is a killed virus vaccine. In most cases, the influenza vaccine causes little to no side effects, but there was a case of a patient who was previously diagnosed with PV and in remission, who later had a flare up following the influenza vaccine on two different occasions (153).

The killed virus anthrax vaccine also had a known case of causing the development of Pemphigus. In this case, the patient received three parts of a six-part vaccine and after each dose, the patient developed new and worsening lesions on the skin and oral cavity (154). In a case report by Berkun et al., a 43-year-old patient who was previously healthy and had no family history of autoimmune disease received the first dose of the recombinant Engerix-B hepatitis B vaccine and three months following the vaccine, was diagnosed with Pemphigus vulgaris (155). It has been proposed that mechanisms by which various vaccines lead to autoimmunity may have common threads. One possibility involves molecular mimicry - vaccine antigens are similar in structure to self-antigens leading to (auto)antibody cross-reactivity. A second proposed mechanism invokes that there is a hyper-immune response to vaccination in certain predisposed individuals that spills into autoimmunity due to an over-exuberant immune state (148, 153).

### 3.6 Sleep

Sleep can be defined in part by a rapid reversible state of immobility and greatly reduced sensory response that is homeostatically regulated (157). The disruption of the circadian rhythm has been conducted in various mammals and sleep has been proven to be vital for survival. Of note, severe deprivation of sleep can lead to a debilitating appearance, increased food intake and/or weight loss, increased energy expenditure, decreased body temperature (158). Sleep is also vital to combat infection.

Sleep architecture has been uncovered with the use of electroencephalographic recordings that trace the electrical patterns of brain activity. The duality of sleep has been explained and divided into non-rapid eye-movement (NREM) sleep and rapid eye-movement (REM) sleep. The two types of sleep have specific characterizations in brain wave variation patterns, eye movements, muscle tone, architecture of sleep and varying effects by sleep regulatory substances (SRS) (159, 160). Cytokines, interleukin 1 β (IL-1) and tumor necrosis factor (TNF)α, growth hormone releasing hormone (GHRH), prolactin and nitric oxide (NO) have been noted for their roles in sleep regulation. The criterion for SRS is as follows: 1) the substance and/or its receptor oscillates with sleep propensity; 2) sleep is increased or decreased with administration of the substance; 3) blocking the action or inhibiting the production of the substance changes sleep; 4) disease states, e.g., infection, associated with altered sleep also change levels of the putative SRS; and finally 5) the substance acts on known sleep regulatory circuits (161–163).

Cytokines are known immunomodulators secreted by specific immune cell types that direct communication between cells. The humoral regulation of sleep by the pro-inflammatory cytokines IL-1 and TNF has been studied by many groups and has linked alterations in sleep to cytokine levels (163–171). A few of these studies support the notion that IL-6 possesses sleep regulatory properties but may not be involved in regulation of spontaneous sleep in healthy animals due to the lack of sleep altering criteria upon antagonization of IL-6 in animals. The effect of IL-1, TNF and IL-6 sleep has been studied in two ways: documented increased expression of regulatory cytokines under physiological and inflammatory conditions versus low-dose and high dose exogenous injection regulatory cytokines. IL-6, TNF and IL-1 are reported to increase NREM sleep in a dose-dependent manner, whereas doses that have shown to maximally increase NREM sleep can suppress REM sleep (164, 165). Of note, in healthy men, IL-6 injections significantly reduce the time spent in REM sleep compared to controls and affect self-reported measures of mood. The alterations in mood and increase in fatigue after injections of IL-6 mirrors symptoms often reported during an infection (166). In addition, in a study of healthy women deprived of sleep for 42 hours, there was a marked increase of TNF, IL-1 and natural killer cell function but no difference in plasma levels of IL-10 (167).

The skin, as well as sleep, both hold unique roles towards combating infection. The skin is the body’s barrier to the external environment and can be greatly affected by changes in sleep. Jang et al. revealed the effects of sleep deprivation on the barrier function of the skin. The restriction of sleep from an average of 8 to 4 hours over 6 consecutive nights significantly decreased the elasticity of the skin (168). In an animal model of psoriasis Hirotsu et al., found that cytokine and humoral levels of proinflammatory cytokines (IL-1 β, IL-6 and IL-12) were significantly increased after sleep deprivation and returned to normal levels after 48h of sleep rebound (169). Disturbances in sleep are known to have profound effects on pain sensitivity, resulting in tenderness and fatigue in healthy individuals. The painful nature of blistering lesions in addition to the cytokine profile in PV point towards a possible bidirectional relationship established between the cytokine-related SRS of sleep and the quality of sleep. While poor sleep quality has been an associated risk factor for various medical disorders that involve immunity and/or autoimmunity (172), the exact relationship of poor sleep quality in patients with pemphigus to the onset and/or exacerbation of disease remains to be elucidated (**Table 7**).

**Table 7:** Studies in support of the hypothesis that PV is triggered or exacerbated by alterations in sleep.

Element	Proposed Pathomechanisms	# of publications in support	Study type	Model	References:
Sleep regulatory cytokines and PV related cytokines (IL-1, TNF, IL-6)	•Increases in IL-1, TNF, and IL-6 with sleep deprivation directly impacts the barrier function of skin (168)	1	Review	–	(158)*, (160, 164), (165)*, (166)*, (168–169, 171)

^*^Indicates a review paper that references literature out of our literature search range (2001-2021).

## 4 Summary and conclusions

There is a substantial amount of data supporting the role of a wide range of environmental and lifestyle factors in the induction and exacerbation of autoimmune diseases including pemphigus vulgaris. ​​In this review, we sought to comprehensively and critically examine the literature to better determine and define the associations between PV and various components constituting the “exposome” such as medications, infections, stress, diet, immunizations, and sleep. It is evident that there is no consensus to what degree each factor can contribute to the onset and course of disease. A myriad of factors may be individually, additively, or synergistically influential.

Our investigation highlights the need for new assessment methods to better track the exposome in real-time in patients across global populations. In particular, longitudinal and prospective data are needed to better understand the shared effects and relationships between multiple genetic and non-genetic (environmental) factors in disease onset or exacerbation. Detailed diaries designed to document large-scale information on patient encounters with environmental elements can then be linked to fluctuating disease activity, response to treatment, and health outcomes, and further linked to genetic variations and immunologic dysregulations to help unravel the intertwined multi-complex factors that conspire to cause and regulate autoimmunity. With the advance and availability of digital medicine devices and services there is the opportunity for app-based platforms, perhaps paired with sensors that monitor environmental as well as biological information, to yield individually curated data collected over time to more critically investigate the interplay of genetics and environment relevant to the autoimmune state. Going further, the impact of socioeconomic class and racial disparities on environment and lifestyle needs to be weighted into the calculus of autoimmune disease risk and prognosis.

**Figure 2:**
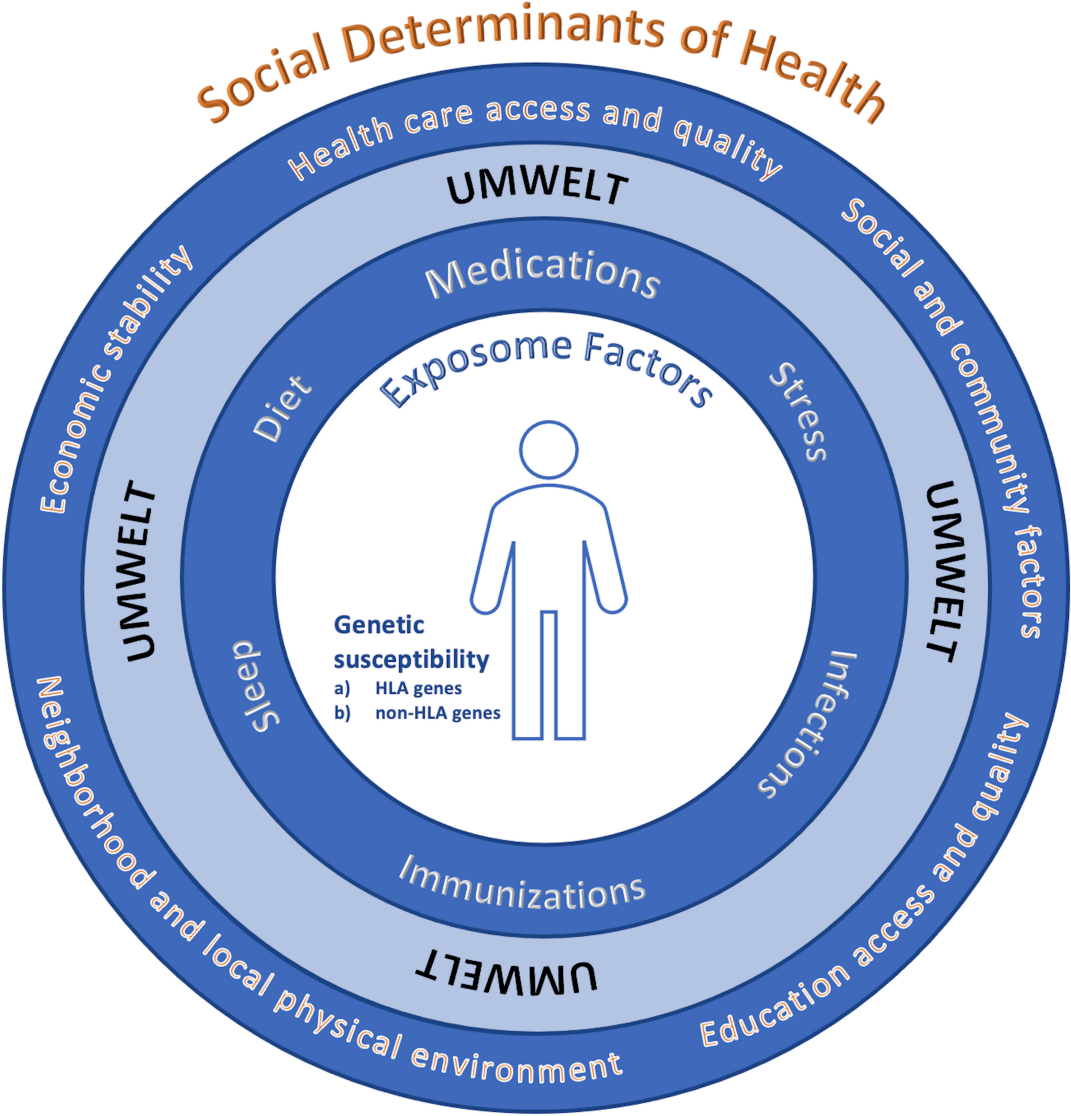
Multifactorial complexities of disease. There is an interplay of: 1) internal genetic factors including **(A)** HLA genes and **(B)** non-HLA genes, and 2) external environmental factors (an individual’s umwelt) that is compromised of both a) exposome factors (including medications, diet, sleep, immunization, infections, stress), and key social determinants of health (SDOH*, including health care access and quality, social and community factors, education access and equality, neighborhood and local physical environment, economic stability) that are inextricably linked and interwoven in a dynamic fashion to set the thresholds for autoimmune susceptibility, disease induction, the severity and course of disease and treatment response. The overall balance and factors is likely to vary across various autoimmune conditions, phenotypic subtypes of disease and the evolution of clinical expression within individuals. *https://health.gov/healthypeople/priority-areas/social-determinants-health.

Overall, we need to arrive at a more detailed mechanistic framework of the multifactorial inputs that determine the set points for immunological self-nonself discrimination at the cellular level. The development of autoimmune disease involves an interplay of both internal genetic factors comprised of HLA genes and non-HLA genes, as well as external environmental factors that collectively can be considered as an individual’s *umwelt* that is compromised of exposome factors (including medications, diet, sleep, immunization, infections, stress) and key social determinants of health (SDOH, including health care access and quality, social and community factors, education access and equality, neighborhood and local physical environment, economic stability), see **Figure 2**. Internal and external factors are inextricably linked and together determine that thresholds and criteria for autoimmune susceptibility, disease induction and disease course. The exact balance of factors are likely to vary across various autoimmune conditions, phenotypic subtypes of disease and the evolution of clinical expression within individuals. Models constructed based on a greater appreciation of the interwoven gene-environment fabric from which disease is formulated could provide investigators, providers, as well as patients with actionable insights and a scientifically rooted rationale to envision increasingly precision based, and ideally, personalized approaches to the prevention and control of autoimmunity.

## Author contributions

OA contributed equally to this work and shares first authorship. DG contributed equally to this work. MA contributed equally to this work. AS conceived of and designed the study. AS contributed equally to this work and shares senior authorship. All authors contributed to the article and approved the submitted version.
